# Significance of pleural effusion detected by metagenomic next-generation sequencing in the diagnosis of aspiration pneumonia

**DOI:** 10.3389/fcimb.2022.992352

**Published:** 2022-12-20

**Authors:** Ling Zhu, Yuqiu Hao, Wei Li, Bingqing Shi, Hongna Dong, Peng Gao

**Affiliations:** Department of Respiratory and Critical Care Medicine, the Second Hospital of Jilin University, Changchun, Jilin, China

**Keywords:** aspiration pneumonia, pleural effusion, mNGS, oral colonization bacteria, diagnosis

## Abstract

**Objective:**

Using metagenomic next-generation sequencing (mNGS) to profile the bacterial pathogen of pleural infection in aspiration pneumonia for therapeutic decision-making.

**Methods:**

Collection and analysis of the clinical and laboratory data of aspiration pneumonia patients who underwent mNGS detection of pleural effusion at the Second Hospital of Jilin University from November 2020 and March 2022.

**Results:**

Nine males and one female were included, aged 33 to 69 years. All patients had chest pain, fever, cough, and hypoxemia symptoms; 90% had expectoration. The laboratory tests revealed that all patients had elevated white blood cell, neutrophil, and C-reactive protein (CRP) levels. Furthermore, erythrocyte sedimentation rate (ESR) increased in 8 patients, and procalcitonin increased in only one patient. Chest CT indicated different degrees of lobar pneumonia and pleural effusion in all patients, and biochemical results implied exudative effusion according to Light criteria. Most routine culture results were negative. Among bacteria identified by mNGS, *Fusobacterium nucleatum* (n=9) was the most common, followed by *Parvimonas micra* (n=7) and *Filifactor alocis* (n=6). Three patients underwent surgical treatment after applying targeted antibiotics, thoracic puncture and drainage, and fibrinolytic septum treatment. After the adjusted treatment, the number of white blood cells, neutrophils, and lymphocytes decreased significantly, indicating the eradication of the infection.

**Conclusions:**

Improving the vigilance of atypical people suffering from aspiration pneumonia is essential. The mNGS detection of pleural effusion clarified the microbial spectrum of aspiration pneumonia, allowing targeted antibiotic administration.

## Introduction

Aspiration is inhaling gross oropharyngeal or gastric contents into the larynx and lower respiratory tract (Neill and Dean, 2019). Aspiration pneumonia is an infection caused by the inhalation of oropharyngeal secretions colonized by pathogenic bacteria or a chemical inflammation caused by the inhalation of aseptic gastric contents (Marik, 2001; Mandell and Niederman, 2019). The diagnosis of aspiration pneumonia can be challenging in the absence of apparent aspiration and other risk factors. When aspiration pneumonia is complicated by pleural effusion, the mortality rates increase compared to those who do not have pleural effusion, making diagnosis and treatment even more crucial for clinicians (Dean et al., 2016). Additionally, prophylactic use of antibiotics in aspiration pneumonitis does not improve outcomes and may drive resistance (Dragan et al., 2018; Neill and Dean, 2019). Conversely, inadequate empirical antibiotic treatment is also an independent contributor to mortality (Maskell et al., 2006). Therefore, improving the diagnostic rate of aspiration pneumonia and understanding the bacteriology of pleural infection in aspiration pneumonia should improve therapeutic decision-making.

Anaerobic bacteria play an important role in oral microbiota (Tanner and Stillman, 1993). However, traditional cultural microbiological methods for isolation and identification can be time-consuming and laborious. Metagenomic next-generation sequencing (mNGS) has been widely used to rapidly identify potential pathogens to reduce improper empirical antibiotic treatment. We conducted a single-center retrospective study to investigate the role of mNGS of parapneumonic pleural effusion in diagnosing and treating the infection in aspiration pneumonia.

## Methods

### Patients and sample collection

This study included ten aspiration pneumonia patients who underwent mNGS detection of pleural effusion during admission to the Second Hospital of Jilin University between November 2020 and March 2022. The clinical data and basic information such as sex, age, height, and weight were extracted from electronic medical records. The laboratory test results such as routine blood, pleural effusion biochemistry, procalcitonin (PCT), C-reactive protein (CRP), erythrocyte sedimentation rate (ESR), comprehensive computed tomography (CT), and arterial blood gas analysis were also collected. Blood samples were cultured for aerobic and anaerobic organisms, but pleural effusion and sputum were only cultured for aerobic organisms. The mNGS procedures, including nucleic acid extraction, library construction, shotgun sequencing by illumination NextSeq 550DX, and bioinformatics analysis, were carried out by WillingMed Technology Co, Ltd (Beijing, China). If the number of qualified reads was greater than or equal to 10, it indicated pathogenic bacteria.

### The mNGS procedures

The mNGS procedures include nucleic acid extraction, library construction, shotgun sequencing by illumination NextSeq 550DX, and bioinformatics analysis. DNA library was prepared by automatic nucleic acid extraction, enzymatic fragmentation, end repair, terminal adenylation, and adaptor ligation. Finished libraries were quantified and normalized by real-time PCR (KAPA) and pooled. Shotgun sequencing was carried out on Illumina NextSeq. Approximately 20 million 50bp single-end reads were generated for each library. During Bioinformatic analysis, sequences of human origin were filtered (GRCh38.p13), and the remaining reads were aligned to a reference database (NCBI nt, GenBank, and in-house curated genomic database) to identify the microbial species and read counts. A negative control (culture medium containing 104 Jurkat cells/mL) was included for each sequencing run. Microbial reads identified from a library were reported if: 1) the sequencing data passed quality control filters (library concentration > 50 pM, Q20 > 85%, Q30 > 80%); 2) negative control (NC) in the same sequencing run does not contain the species or the RPM (sample)/RPM (NC) ≥ 5, which was determined empirically as a cutoff for discriminating true-positives from background contaminations.

### Ethics statement

This study was approved by the Ethics Committee of the Second Hospital of Jilin University (approval number: 2022-186). Written informed consent was exempted from ethical review as it was a retrospective study and patient data were anonymized.

### Statistical analyses

We used the Shapiro-Wilk test to assess whether quantitative variables were normally distributed. We present normally distributed continuous variables as mean ± standard deviation. Non-normally distributed continuous variables are presented as the median and interquartile range (IQR). Categorical variables are presented as counts and percentages. For laboratory results, we also assessed whether there were differences before and after treatment. Paired-samples T-test and Wilcoxon signed-rank test were used for normally and non-normally distributed continuous variables, respectively. A P-value below 0.05 was defined as significant. We used SPSS (version 22.0) and Graphpad Prism (version 8.0) for all analyses.

## Results

### Clinicopathological information

Nine males and one female were included, aged between 33 and 69 years (average age of 53.4 years). The BMI was between 21.38 and 28.9 (average 23.856), with two values exceeding 25. As shown in **Table 1**, four patients had hypertension, two had previous cerebral infarction, one had bronchiectasis, one had diabetes, one had coronary heart disease, one had obsolete pulmonary tuberculosis, and the other four were in good health. Five patients had a long-term history of smoking, and four had a long-term history of drinking, with one drinker reporting increased chest pain after drinking and one vomiting during the disease. On admission, all patients had chest pain, fever, and cough, with most of them having expectoration and only one having a dry cough. Two patients gradually developed malodorous sputum during the disease. One patient had hemoptysis, and two had chills before the fever. All patients had hypoxemia, as determined by arterial blood gas analysis.

**Table 1:** Patients’ characteristics upon admission.

	Patients (n=10)
Basic information	
Male	9 (90%)
Smoking	5 (50%)
Drinking	4 (40%)
Underlying disease	
Hypertension	4 (40%)
Cerebral infarction	2 (20%)
Diabetes	1 (10%)
Bronchiectasis	1 (10%)
Coronary heart disease	1 (10%)
Obsolete pulmonary tuberculosis	1 (10%)
**No underlying disease**	4 (40%)
Clinical symptoms	
Fever	10 (100%)
Chest pain	10 (100%)
Cough	10 (100%)
Smelly sputum	2 (20%)
Chills	2 (20%)
Hypoxemia	10 (100%)
Laboratory index	
Positive blood culture results	0( 0%)
Positive pleural effusion results	0 (0%)
Positive sputum culture results(Candida albicans)	4 (40%)
Location of the pulmonary inflammation	
Parapneumonic effusion	10 (100%)
Hydropneumothorax	2 (20%)
Empirical anti-infective treatment	
Carbapenem (Meropenem, Ertapenem)	10 (100%)
Oxazolidinone (Linezolid)	1 (10%)
Nitroimidazole (Ornidazole)	2 (20%)
β-lactamase inhibitors	1 (10%)
Treatment	
Placing the drainage tube	10 (100%)
Fibrinolysis therapy	10 (100%)
Surgical treatment	3 (30%)

Data are presented as n (%). Fever: axillary temperature is greater than or equal to 37.3 degrees Celsius; Smoking: The average amount of smoking is greater than or equal to 10 packs per year; Drinking: Average daily consumption of more than or equal to 20 ml of pure alcohol; Hypoxemia: When the patient breathed 20.9% oxygen at sea level, the arterial oxygen partial pressure was lower than 80mmHg.

### Laboratory tests

**Figure 1:**
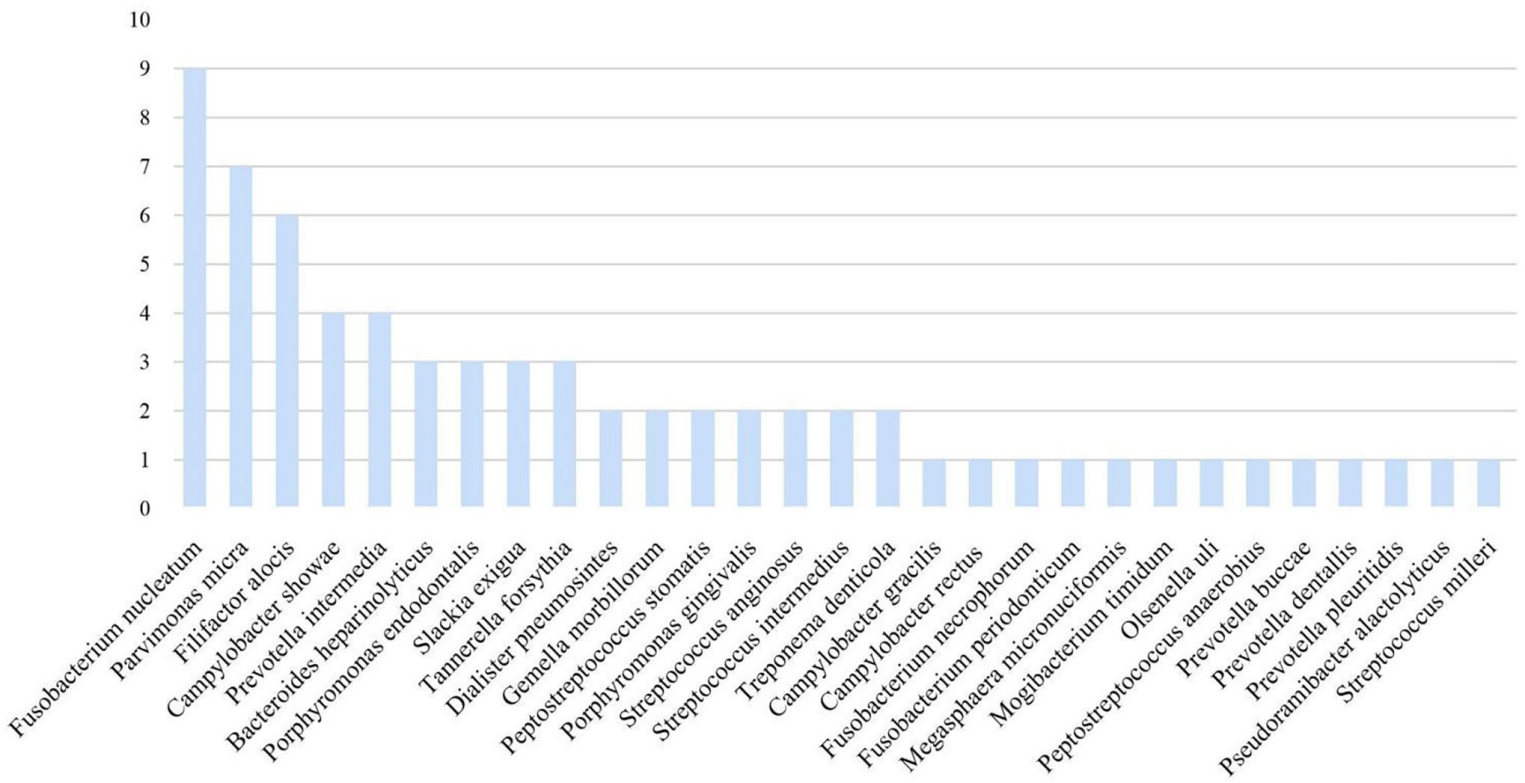
Species distribution of pathogens detected by pleural effusion mNGS.

**Figure 2:**
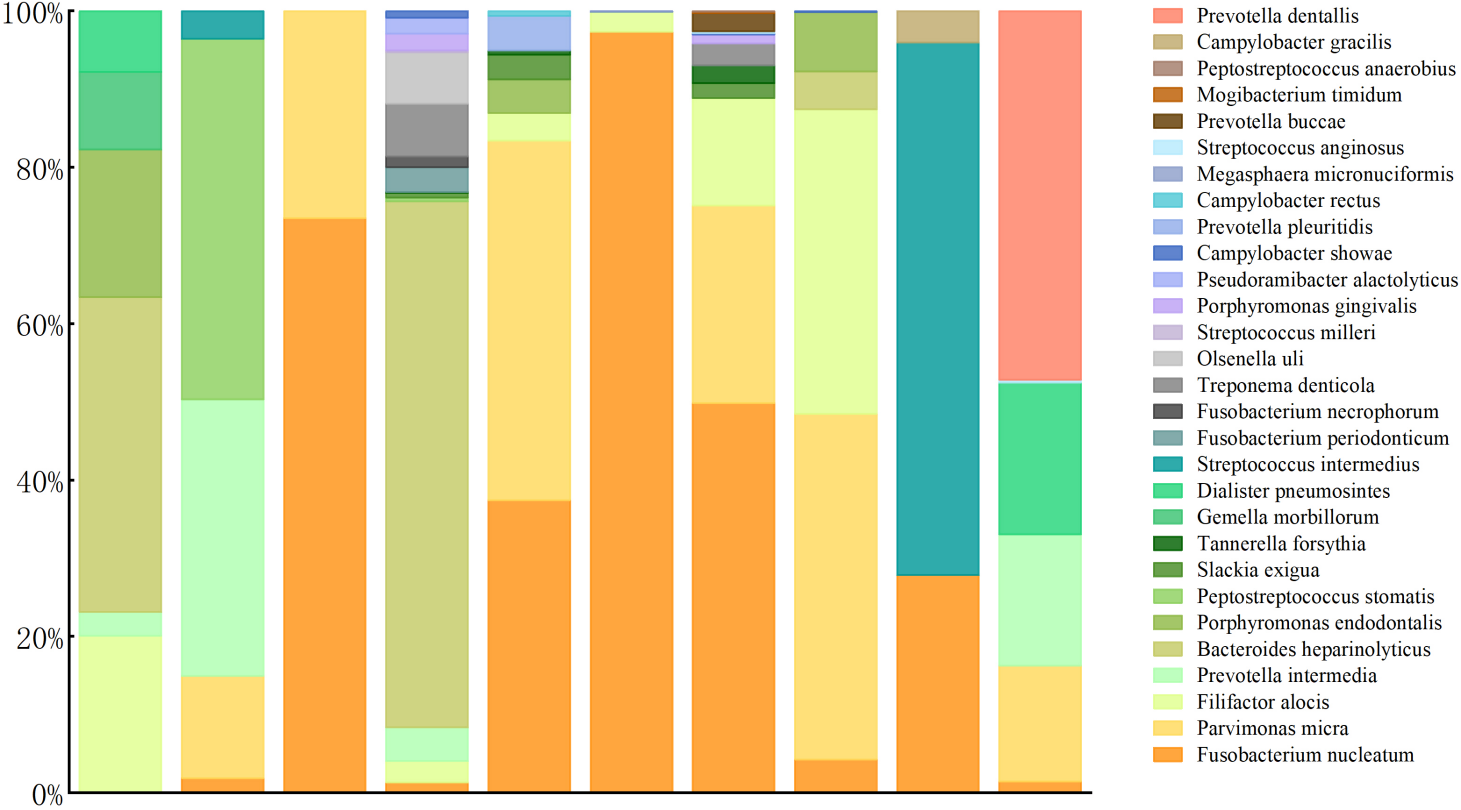
Species distribution and relative abundance of pathogens detected by pleural effusion mNGS.

The laboratory tests revealed elevated white blood cell, neutrophil, and CRP levels in all patients. Furthermore, ESR increased in eight patients (the other two were not tested), and procalcitonin increased in only one. All patients underwent therapeutic thoracocentesis drainage. All biochemical test results implied exudative effusion according to Light criteria. Chest CT indicated different degrees of lobar pneumonia and pleural effusion in all patients, with two having hydropneumothorax. The blood cultures of seven patients and blood mNGS of two patients were negative. All patients had sputum smears, sputum culture, and pleural effusion culture; the results of four patients’ sputum cultures showed *Candida albicans*. The patients did not undergo bronchoscopy for personal or physical reasons (**Table 1**, **Table 2**). The patients’ pleural effusion samples were sent for mNGS, which reported oral colonization bacterial infection. **Figure 1** shows the types of oral colonization bacteria. Among pathogens detected by mNGS, *Fusobacterium nucleatum* (n=9) was the most common bacteria, followed by *Parvimonas micra* (n=7) and *Filifactor alocis* (n=6). **Figure 2** depicts in detail the mNGS results that revealed the relative abundance of various pathogens in each patient’s pleural effusion.

**Table 2:** Patients’ characteristics and laboratory findings upon admission.

	SD	IQR
Basic situation		
Age	53.40 ± 13.11	
Height(m)	1.72 ± 0.06	
Weight(kg)	71.30 ± 14.08	
BMI	23.86 ± 3.70	
Laboratory index		
WBC × 10^9^/L		12.85(11.40,16.40)
NEU × 10^9^/L		10.39(9.07,13.95)
LYM × 10^9^/L	1.37 ± 0.48	
MON × 10^9^/L	1.11 ± 0.31	
HB(g/L)	129.30 ± 24.72	
PLT × 10^9^/L	322.2 ± 121.88	
Serum total protein(g/L)	66.41 ± 8.57	
Serum LDH(U/L)	162.70 ± 31.19	
Pleural effusion total protein (g/L)		53.35(44.63,57.23)
Pleural effusion LDH(U/L)		1443.50(979.50,3367.75)
Pleural glucose level(mmol/l)		0.73(0.07,2.26)
Pleural chlorine(mmol/l)	98.48 ± 4.72	
Pleural WBC count(10^6^/L)		3013.50(1308.00,28048.50)
ADA(U/L)		31.135(22.92,93.89)
CRP(mg/L)	56.04 ± 10.21	
PCT(ng/ml)		0.401(0.16,0.70)
ESR(mm/s)	68.75 ± 16.32	
**Hospitalization days**		19.00(12.5,23.25)
**The days of the disease**		26.5(22.5,32.25)

Data are presented as mean (SD) or median (IQR). WBC, white blood cell; NEU, neutrophils; LYM, lymphocyte; MON, mononuclear cell; HB, hemoglobin; PLT, platelets; PCT, procalcitonin; CRP, C-reactive protein; ESR, erythrocyte sedimentation rate; BMI, body mass index; LDH, lactate dehydrogenase; ADA, adenosine deaminase; ESR, erythrocyte sedimentation rate.

### Treatments and outcome

**Figure 3:**
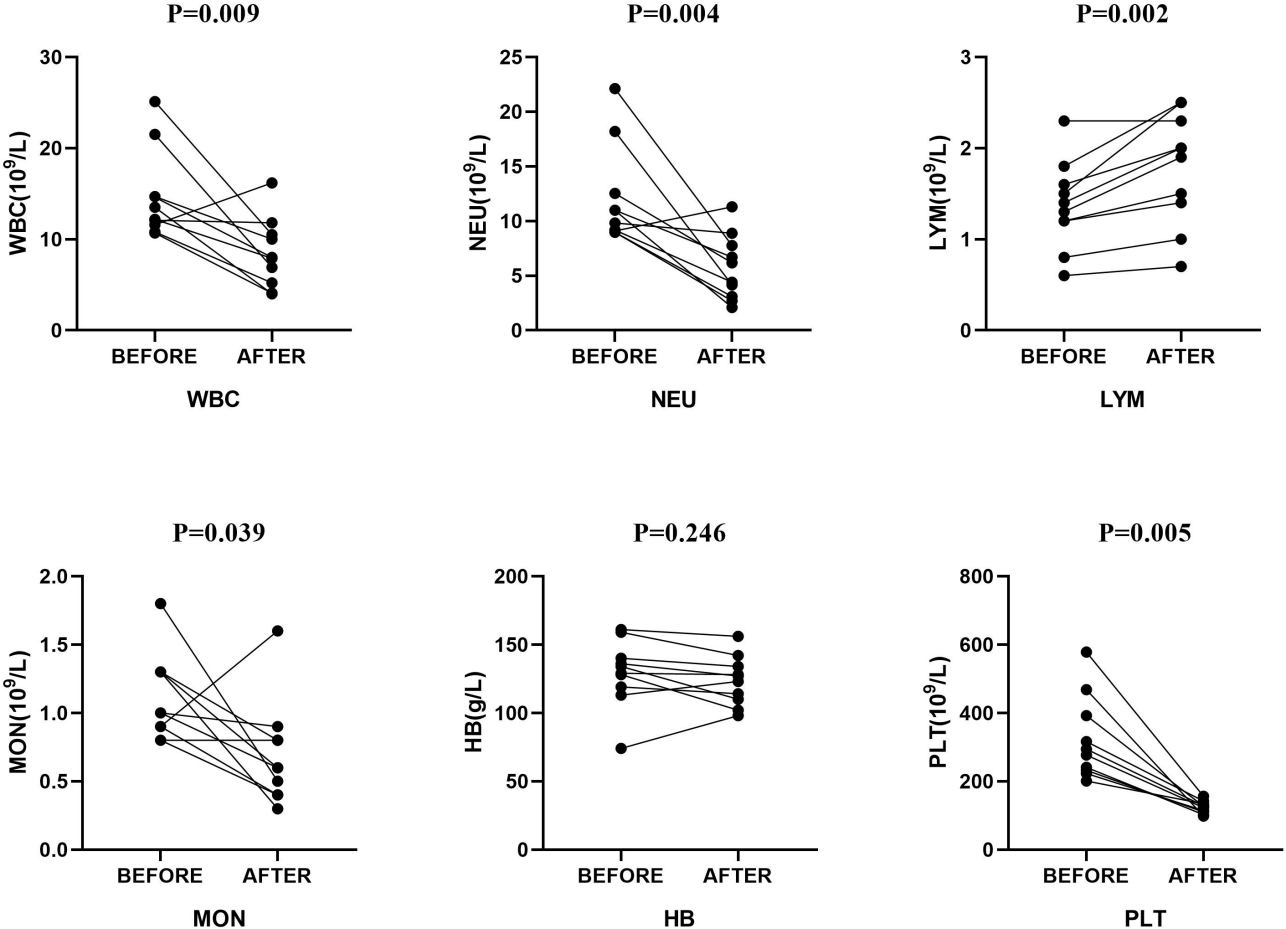
Comparison of laboratory indices before and after treatment. P values are presented for the Mann-Whitney test and the t-test for paired samples. WBC, white blood cell; NEU, neutrophils; LYM, lymphocyte; MON, mononuclear cell; HB, hemoglobin; PLT, platelets.

**Figure 4:**
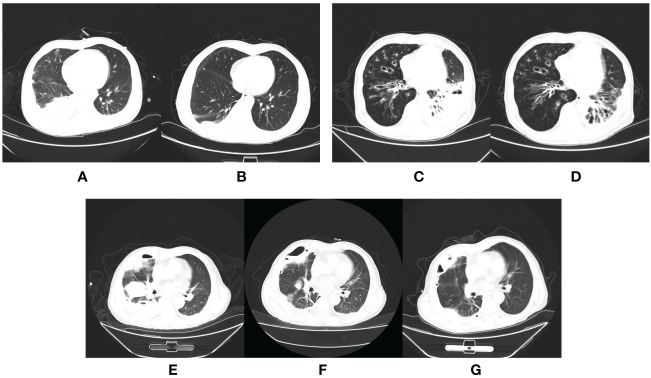
The CT scan of aspiration pneumonia. Chest CT of a patient who was in good health showed moderate right-sided pleural effusion on the day of admission **(A)** and a significantly reduced, small amount of right-sided pleural effusion before discharge from the hospital **(B)**. Chest CT of a patient with hypertension, and previous cerebral infarction showed inflammation of the left lower lobe of the lung on admission **(C)** and partial absorption after targeted antibiotic treatment for two months **(D)**. Chest CT of a patient showed a large area of inflammation and a partial cavity in the right lung on the day of admission **(E)**; the inflammation was partially absorbed, but the cavity became larger after targeted antibiotic treatment **(F)**, and smaller after absorption of inflammation postoperatively **(G)**.

Upon admission, all patients received empirical anti-infective treatment. As shown in **Table 3**, all patients received carbapenem, seven received additional moxifloxacin (1 patient was considered a case of tuberculous pleural effusion at that time), two patients received additional ornidazole, and one received linezolid for suspected drug-resistant bacterial septicemia. At the same time, each patient received multiple intrapleural irrigations with urokinase and gentamicin. After reviewing the pleural effusion mNGS results, aspiration pneumonia in these patients was confirmed, and antibiotic treatments were adjusted. In addition to the two patients who had already received ornidazole at the time of admission, six of the remaining eight received additional ornidazole. Of the last two patients, one patient’s primary care physician continued treatment with meropenem considering its ability to cover anaerobic bacteria, and the other underwent surgery for prolonged fever, during which a beta-lactamase inhibitor was administered. Seven patients’ imaging and clinical manifestations gradually improved compared to those at admission. The remaining two patients without apparent improvement (including one without ornidazole) were eventually discharged after surgical treatment. **Figure 3** compares laboratory indices before and after the adjusted treatments. **Figure 4** shows the patient’s chest CT from admission to discharge. The number of white blood cells, neutrophils, and lymphocytes decreased significantly, absorption of pulmonary inflammation, and decreased pleural effusion, indicating the infection’s eradication.

**Table 3:** Antibiotics used before and after mNGS.

Patient	mNGS results	Antibiotics		Surgical treatment	Hospitalization days	Outcomes
		Before mNGS	After mNGS			
1	Oral anaerobic bacteria	Carbapenem, Quinolones	Carbapenem, Nitroimidazole	No	14	Improved
2	Oral anaerobic bacteria	Carbapenem, Nitroimidazole	Carbapenem, Nitroimidazole	No	21	Improved
3	Oral anaerobic bacteria	Carbapenem, Quinolones	Carbapenem, Nitroimidazole	No	9	Improved
4	Oral anaerobic bacteria	Carbapenem, Oxazolidinone, and Quinolones	Carbapenem, Nitroimidazole	No	17	Improved
5	Oral anaerobic bacteria	β-lactamase inhibitors, Nitroimidazole	Carbapenem, Nitroimidazole	No	11	Improved
6	Oral anaerobic bacteria	Carbapenem, Quinolones	Carbapenem, Nitroimidazole	No	23	Improved
7	Oral anaerobic bacteria	Carbapenem, Quinolones	Carbapenem, Nitroimidazole	Yes	24	Improved
8	Oral anaerobic bacteria	Carbapenem, Nitroimidazole	Carbapenem, Nitroimidazole	No	24	Improved
9	Oral anaerobic bacteria	Carbapenem, Quinolones	Carbapenem	Yes	45	Improved
10	Oral anaerobic bacteria	Carbapenem, Quinolones	β-lactamase inhibitors	Yes	13	Improved

mNGS, metagenomic next-generation sequencing.

## Discussion

Traditionally, a characteristic clinical history (witnessed macro aspiration), risk factors, and compatible findings on chest radiography constitute the diagnostic criteria of aspiration pneumonia (Mandell and Niederman, 2019). However, in most cases, patients with aspiration pneumonia lack an apparent history of aspiration (Huxley et al., 1978). Risk factors for aspiration include advanced age, oropharyngeal dysphagia, oral bacterial colonization, gastroesophageal disorders, neurological disorders, stroke, altered mental status, use of sedatives, hypnotics, or psychotropic drugs, obstructive sleep apnea, and other special circumstances (Beal et al., 2004; Almirall et al., 2021). Studies have shown that all healthy individuals frequently inhale pharyngeal secretions during deep sleep (Almirall et al., 2021), and whether this results in lung infection depends on the host’s defense mechanism. But it is difficult to distinguish bacterial aspiration pneumonia from chemical aspiration pneumonia by clinical manifestations alone. Previous studies have used alpha-amylase levels, bronchoalveolar lavage cultures, and serum procalcitonin levels to distinguish infectious pneumonia from non-infectious pneumonia (El-Solh et al., 2011; Weiss et al., 2013).Nevertheless, these methods failed to produce the desired results. Furthermore, the imaging findings, usually related to gravity, could not clearly show the condition, probably because of the patients’ activities during the disease or the variable use of antibiotics before admission. The factors mentioned above can easily lead to misdiagnosis and missed diagnosis of aspiration pneumonia or difficulty determining whether it is infectious aspiration pneumonia.

Sputum smears and cultures have traditionally been used to identify the infectious pathogen of aspiration pneumonia, but sampling from the microbial-rich nasal cavity or distal airways of the mouth through the pharynx is prone to contamination. Some studies have used bronchoalveolar lavage fluid (BALF) culture to distinguish bacterial pneumonia from non-infectious pneumonia (chemical and mild aspiration pneumonia) (Ferreiro et al., 2015). When pleural effusion complicates aspiration pneumonia, the mortality rate increases. Without appropriate antibiotic treatment, a simple parapneumonic effusion (SPPE) may progress to a fibrinopurulent stage (complicated parapneumonic effusion or empyema) with low pH (<7.20), low glucose levels (<60 mg/dl) and high lactate dehydrogenase (LDH), indicating bacterial migration to the chest’s pleural space, a severe condition associated with increased morbidity and mortality (Sahn, 2007). At this time, pleural effusion culture is often used. However, infectious aspiration pneumonia is usually caused by oral colonization anaerobes, such as *Parvimonas micra*, *Fusobacterium nucleatum*, *Prevotella intermedia*, *Prevobacter*, and *Streptococcus intermedius* (Scannapieco, 1999), which can cause pleural effusion (Cobo et al., 2017; Brennan and Garrett, 2019; Galliguez et al., 2021). Anaerobes are difficult to culture and isolate because their fastidious nature requires specific inoculation methods and culture equipment.

Moreover, the culture requires a long time, and the clinical antibiotic adjustment feedback lags behind. As a new pathogen detection method, mNGS has been gradually introduced into clinical practice, outperforming conventional detection methods (Huang et al., 2021). Lately, mNGS has provided a promising means for pathogen-specific diagnosis and enhanced diagnostic strategy for lower respiratory tract infections. mNGS can be used in a wide range of specimen types (sputum, throat swab, blood, alveolar lavage fluid, pleural fluid), and it can directly carry out high-throughput sequencing of nucleic acids in clinical samples without distinction and selection (Zheng et al., 2021). Because human lungs are not entirely aseptic (Huffnagle et al., 2017; Cookson et al., 2018), the mNGS of BALF sampled is inevitably disturbed by the normal colonization of microorganisms. In contrast, the pleural cavity has less background miscellaneous bacteria, resulting in less interference and higher accuracy in interpreting mNGS results. In addition, patients experienced less trauma during diagnostic thoracic puncture than during bronchoscopy alveolar lavage.

In the present study, the mNGS detection of pleural effusion clarified the microbial spectrum, confirming the diagnosis of infectious aspiration pneumonia and facilitating the administration of targeted antibiotics. Ornidazole effectively treats anaerobic infection (Pradeep et al., 2012). Anaerobes often coexist with aerobes, so effective antibiotics against both infectious pathogens were used, such as carbapenem antibiotics and quinolone antibiotics (Brook, 2011; Noguchi et al., 2021). It is worth noting that patients who do not receive ornidazole have a higher rate of surgery, but more data are needed to validate this finding. The recurrent fever, leukocytosis and/or elevated C-reactive protein (CRP), and a considerable amount of residual pleural effusion, revealed by radiological examinations, always indicate the failure of fibrinolysis therapy. At this time, surgical intervention should be considered without further delay to improve the survival rate of patients (Ferreiro et al., 2015), In this study, the white blood cell count, neutrophil count, CRP, ESR, and other indices were significantly lower than those at admission, and the total leukocyte count in one patient was higher than that before admission; considered the postoperative stress state.

This study has several limitations. First, the small sample size could have introduced some degree of bias. Second, because this was a retrospective study, some clinical indicators were missed. As a result, comparing the before and after changes in some indicators, such as the nature of the pleural effusion, the level of ESR, and so on, in some patients is difficult. Moreover, due to the high sensitivity of mNGS, its specificity is lower than that of traditional cultural microbiological methods, necessitating validation tests for mNGS. The ability of mNGS to differentiate pathogens from normal microorganisms, and environmental pollutants to guide diagnosis and treatment is a challenge for future clinical research (Han et al., 2019). To compensate for the aforementioned limitations, prospective follow-up research is worthwhile.

## Conclusions

Even without a history of aspiration, risk factors, and typical imaging findings, patients with acute chest pain, fever, exudative pleural effusion, elevated leukocytes, CRP, and ESR levels should have a low threshold for aspiration pneumonia diagnosis. Due to its high sensitivity, specificity, and less trauma, mNGS detection of pleural effusion has a good application prospect in the accurate diagnosis of aspiration pneumonia for improving the prognosis of patients. However, it has some shortcomings, such as high cost and false-negative results in simple parapneumonic effusion. Therefore, reasonable standardized clinical guidelines and protocols should be produced to promote mNGS use in clinical practice.

## Data availability statement

The data presented in this study can be found in online repository. The name of the repository is NCBI Sequence Read Archive(SRA) database, and accession number is PRJNA910987.

## Ethics statement

The studies involving human participants were reviewed and approved by the Ethics Committee of the Second Hospital of Jilin University. Written informed consent for participation was not required for this study in accordance with the national legislation and the institutional requirements.

## Author contributions

PG contributed to the conception of the study. LZ and YH drafted the manuscript. WL, BS, and HD reviewed and revised it critically for important intellectual content. All authors revised the manuscript critically and approved the final version.
